# Changes in Functional Performance During and After a Supervised Exercise Programme in People with Multiple Sclerosis: A Secondary Uncontrolled Longitudinal Study

**DOI:** 10.3390/life16101630

**Published:** 2026-09-29

**Authors:** Filippo Tarditi, Luca Beratto, Francesco Borrelli, Emanuele Koumantakis, Giulia Actis Dato, Gaetano Rossi, Lara Bressy, Elena Turiano, Alberto Rainoldi, Andrea Silvio Benso, Anna Mulasso

**Affiliations:** 1 Department of Medical Sciences, University of Torino, 10126 Torino, Italy; luca.beratto@unito.it (L.B.); francesco.borrelli@unito.it (F.B.); giulia.actisdato@unito.it (G.A.D.); gaetano.rossi@unito.it (G.R.); alberto.rainoldi@unito.it (A.R.); andrea.benso@unito.it (A.S.B.); anna.mulasso@unito.it (A.M.); 2 NeuroMuscular Function|Research Group, School of Exercise and Sport Sciences (SUISM), University of Torino, 10126 Torino, Italy; lara.bressy.lb@gmail.com; 3 Center for Biostatistics, Epidemiology, and Public Health, Department of Clinical and Biological Sciences, University of Torino, 10126 Torino, Italy; emanuele.koumantakis@unito.it; 4 Cancer Epidemiology Unit, Città della Salute e della Scienza di Torino University Hospital, 10126 Torino, Italy; 5 160CM APS, 10126 Torino, Italy; elena.turiano@gmail.com; 6 Division of Endocrinology, Diabetology and Metabolism, Department of Medical Sciences, University of Torino, 10126 Torino, Italy

**Keywords:** multiple sclerosis, supervised exercise, high-intensity interval training, resistance training, physical exercise, exercise capacity, functional performance, follow-up

## Abstract

Background: Exercise is clinically relevant for people with multiple sclerosis (PwMS), but participation in structured programmes is often intermittent. This exploratory study described changes in functional and exercise-related outcomes during and after a supervised exercise programme. Methods: This secondary uncontrolled longitudinal analysis included 20 adults with MS who had three consecutive assessments: before (T1) and after (T2) a 12-week programme comprising two weekly 60-min supervised sessions, and after an approximately three-month period without supervised training. Outcomes included functional performance, gait parameters, fatigue and exercise-testing outcomes. Linear mixed-effects models were used as the main longitudinal analysis, with paired Wilcoxon tests as complementary comparisons. Results: The median 2-Minute Walk Test distance increased from 121 m at baseline to 126 m after the programme and was 125 m at follow-up. The corresponding mixed-model estimate for T2 versus T1 was 5.00 m (95% CI: −0.29 to 10.29; *p* = 0.063), whereas the paired comparison yielded *p* = 0.046. Achieved workload, VO_2_ estimated using the FRIEND equation, and percentage of predicted VO_2_ achieved showed numerical increases after the programme, but these changes were not statistically significant. Most other outcomes remained broadly stable, with heterogeneous individual trajectories. Conclusions: Modest within-group changes were observed in selected walking and exercise-test-derived outcomes, but the findings were not consistently supported across statistical approaches. Given the small sample and absence of a control group, the results should be considered exploratory and cannot establish programme effectiveness or programme-related preservation of function.

## 1. Introduction

Multiple sclerosis (MS) is a chronic inflammatory and neurodegenerative disease of the central nervous system, affecting over 2.3 million people worldwide and representing one of the leading causes of non-traumatic disability in young adults [1,2,3]. Beyond the primary neurological manifestations related to demyelination and neurodegeneration, people with multiple sclerosis (PwMS) frequently experience fatigue, mobility impairment, balance dysfunction, deconditioning, reduced exercise capacity, and impaired quality of life [4,5]. These manifestations may interact with each other and contribute to progressive limitations in independence and daily functioning.

For many years, PwMS were discouraged from exercising because of concerns regarding symptom exacerbation, particularly in relation to heat sensitivity [6,7]. This view has now changed substantially: physical activity and exercise are regarded as safe and clinically relevant components of MS care, with evidence supporting beneficial effects on aerobic capacity, muscle strength, balance, mood, fatigue, and quality of life, without increasing relapse risk [8,9,10]. Accordingly, exercise-based rehabilitation is increasingly recognized as an essential component of multidisciplinary MS management.

Despite these benefits, physical inactivity remains highly prevalent among PwMS. Up to 78% of individuals with MS have been reported to engage in no physical activity, compared with approximately 38% of age-matched individuals in the general population [6]. This inactivity is clinically relevant because PwMS often present with reduced aerobic capacity, with VO_2_max values approximately 30% lower than those of healthy controls, together with reduced maximal work rate and muscle strength, particularly in the lower extremities [7,11,12]. Slower gait velocity, altered muscle composition, increased resting heart rate in some individuals, and a reduced rate of force development have also been reported [7]. These impairments may contribute to functional decline and further discourage engagement in physical activity, thereby creating a cycle of deconditioning and diminished functional reserve.

Several barriers may explain the difficulty of maintaining regular exercise in this population, including fatigue, symptom fluctuations, low self-efficacy, transportation difficulties, comorbidities, environmental constraints, reduced motivation, and limited access to tailored programmes [11,12,13,14]. As a result, exercise participation in real-world MS care is often intermittent rather than continuous. Patients may alternate periods of supervised training with periods of autonomous activity, reduced training exposure, or complete interruption. Understanding the trajectory of functional outcomes following a supervised exercise programme is therefore clinically relevant. In MS, the role of exercise extends beyond the achievement of short-term adaptations and may also include the preservation of functional capacity and the prevention of inactivity-related deconditioning.

Among exercise modalities, moderate-intensity aerobic and resistance training have been extensively studied in PwMS. More recently, high-intensity interval training (HIIT), alone or combined with resistance training, has attracted increasing interest as a time-efficient approach with the potential to provide complementary benefits for cardiovascular and musculoskeletal function [11,15]. Preliminary evidence suggests that combined HIIT and resistance training may positively influence systemic markers, including oxidative stress, inflammatory profile, lipid metabolism, and body composition [16]. Network meta-analytic evidence has indicated that HIIT may be among the most effective exercise modalities for improving muscular fitness, although its effects on fatigue may differ from those of mind-body exercise or resistance-based interventions [17]. Similarly, a recent meta-analysis reported beneficial effects of HIIT on exercise capacity and fatigue in PwMS [18].

A key issue in this context is heterogeneity of response. Baseline disability, commonly assessed using the Expanded Disability Status Scale (EDSS), may influence both absolute performance and the potential for change. Individuals with lower disability may exhibit better baseline performance and potential ceiling effects in some functional tests, whereas those with higher disability may have greater room for measurable improvement but also greater limitations to training tolerance. Similarly, adherence may influence the persistence of exercise-related benefits, although it may also reflect broader clinical, behavioural, and motivational factors rather than exercise exposure alone.

The present exploratory study aimed to describe within-group changes in functional performance, fatigue, physical activity, gait, and exercise-test-derived capacity in PwMS assessed before and immediately after a supervised exercise programme and after an approximately three-month period without supervised training. Additional exploratory analyses examined whether longitudinal trajectories differed according to baseline EDSS and adherence to the supervised programme. Given the uncontrolled study design, the analyses were intended to characterize changes over time rather than establish the causal effectiveness of the intervention.

## 2. Materials and Methods

### 2.1. Study Design and Participants

This study was a secondary longitudinal analysis of routinely collected standardized data from supervised exercise programme for PwMS.

Participants underwent assessments before and after a 12-week supervised exercise programme. For the present analysis, eligible participants were unique individuals for whom three consecutive standardized assessments were available: an assessment before a supervised exercise programme (T1), an assessment at the end of the programme (T2), and a subsequent assessment performed approximately three months later, before the beginning of a new supervised programme (FU). Each participant contributed only one sequence of observations to the analysis.

The analytical sample therefore consisted of participants with sufficient consecutive observations to describe an individual longitudinal trajectory. Other individuals may have participated in one or more exercise programmes but were not eligible for the present analysis if three consecutive assessments were not available. The assessments included in the present analysis were conducted between February 2024 and February 2025.

The analysis was exploratory and was intended to characterize longitudinal trajectories across clinically relevant functional and exercise-related outcomes rather than to test a single prespecified confirmatory hypothesis.

Eligible participants were identified and enrolled by the clinical team among members of 160CM APS attending the Neurology Unit of the Città della Salute e della Scienza, Torino. We enrolled adults aged 18 years or older with a confirmed diagnosis of MS, clinically stable disease, and an Expanded Disability Status Scale (EDSS) score between 1.0 and 6.0, corresponding to the ability to ambulate independently with or without walking aids. Clinical stability was defined as the absence of an acute MS relapse or clinically relevant neurological deterioration during the three months preceding enrolment in the exercise programme. Participants were also required to be able to perform exercise on a stationary cycle ergometer. All participants had undergone a neurological evaluation within two months before enrolment, including EDSS assessment by a neurologist. Exclusion criteria were unstable cardiovascular, pulmonary, metabolic, neurological, or musculoskeletal conditions contraindicating exercise participation; pregnancy; moderate-to-severe cognitive impairment limiting the ability to understand instructions or provide reliable cooperation during testing and training; inability to safely participate in the supervised exercise programme; or any other clinical condition judged by the study physicians to interfere with participation. Outcome assessments were performed as part of routine clinical practice. Assessors were not blinded to the assessment timepoint, as evaluations were conducted sequentially within each exercise programme.

All participants provided written informed consent before study procedures. The study was conducted in accordance with the Declaration of Helsinki and received approval from the Bioethics Committee of University of Turin [protocol code #0218920 of 12 April 2023].

Figure 1 Study design and assessment timeline. Study design and assessment timeline. Participants underwent clinical, functional, and exercise-testing assessments at baseline (T1), repeated the same assessments after 12 weeks of supervised training (T2), and were reassessed after approximately three months without supervised exercise (follow-up). The intervention consisted of two weekly supervised sessions including HIIT, resistance training, flexibility, and balance exercises.

### 2.2. Medical Evaluation and Exercise Eligibility

Before training participation, all patients underwent a pre-exercise medical evaluation to assess safety, exercise eligibility, and individualized exercise prescription. The evaluation included medical history, review of the most recent neurological assessment, physical examination with specific attention to muscle strength and peripheral musculoskeletal limitations, cardiovascular examination, resting electrocardiogram, cardiovascular risk stratification, and a symptom-limited exercise test on an electronically braked cycle ergometer. Study timeline is shown in Figure 1.

### 2.3. Exercise Testing and Derived Variables

The maximal symptom-limited exercise test was performed on an electronically braked cycle ergometer (Cosmed Srl, Rome, Italy) using an individualized ramp protocol. The ramp was tailored according to the participant’s expected exercise capacity, functional limitations, and clinical condition, with the aim of achieving an exercise phase lasting approximately 8–12 min, according to the Wasserman equation and reduced by approximately one-third in sedentary or markedly deconditioned individuals [19]. Participants were instructed to maintain a cadence of approximately 70 revolutions per minute.

The test was supervised and interpreted by trained physicians. Heart rate, electrocardiographic findings, workload, oxygen saturation, symptoms, and rating of perceived exertion using the Borg scale were monitored throughout the test. The highest completed workload was recorded as peak achieved workload. The test was terminated at volitional exhaustion or in the presence of peripheral muscle fatigue, inability to maintain the target cadence, participant request, clinically relevant arrhythmias, oxygen desaturation, or other findings considered incompatible with safe continuation.

Respiratory gas exchange analysis was not performed. Therefore, the VO_2_ variables reported in this study represent estimates derived from the achieved workload and reference equations rather than directly measured VO_2_peak or VO_2_max.

For the present analysis, exercise-test-derived variables included peak achieved workload, VO_2_ estimated from the achieved workload using the FRIEND equation, Wasserman–Hansen-predicted VO_2_, and the percentage of predicted VO_2_ achieved [20].

VO_2_ corresponding to the achieved cycle-ergometer workload was estimated using the FRIEND cycle-ergometry equation:

FRIEND-estimated VO_2_ (mL·kg^−1^·min^−1^) = 1.74 × [(peak workload in W × 6.12)/body mass in kg] + 3.5.

The expected VO_2_ was calculated using the sex-specific Wasserman–Hansen equations, based on age, height, actual body mass, and predicted body mass [19,21].

For men:

Cycle factor = 50.72 − (0.372 × age in years).

Predicted body mass (kg) = (0.79 × height in cm) − 60.7.

When actual body mass was lower than predicted body mass:

Predicted VO_2_ (mL/min) = [(predicted body mass + actual body mass)/2] × cycle factor.

When actual body mass was equal to predicted body mass:

Predicted VO_2_ (mL/min) = actual body mass × cycle factor.

When actual body mass was greater than predicted body mass:

Predicted VO_2_ (mL/min) = (predicted body mass × cycle factor) + [6 × (actual body mass − predicted body mass)].

For women:

Cycle factor = 22.78 − (0.17 × age in years).

Predicted body mass (kg) = (0.65 × height in cm) − 42.8.

When actual body mass was lower than predicted body mass:

Predicted VO_2_ (mL/min) = [(predicted body mass + actual body mass + 86)/2] × cycle factor.

When actual body mass was equal to predicted body mass:

Predicted VO_2_ (mL/min) = (actual body mass + 43) × cycle factor.

When actual body mass was greater than predicted body mass:

Predicted VO_2_ (mL/min) = [(predicted body mass + 43) × cycle factor] + [6 × (actual body mass − predicted body mass)].

The resulting absolute predicted VO_2_, expressed in mL/min, was divided by actual body mass to obtain the relative Wasserman–Hansen-predicted VO_2_ in mL·kg^−1^·min^−1^.

The percentage of predicted VO_2_ achieved was calculated as follows:

Percentage of predicted VO_2_ achieved = (FRIEND-estimated VO_2_/Wasserman–Hansen-predicted VO_2_) × 100.

Age-predicted maximal heart rate was calculated as 220 − age in years. The percentage of maximal heart rate achieved was calculated as: (peak heart rate recorded during the test/age-predicted maximal heart rate) × 100 [22].

Thus, the three exercise-test-derived variables represent distinct but mathematically related measures: VO_2_ estimated from the achieved workload using the FRIEND equation, expected VO_2_ derived from the Wasserman–Hansen reference equations, and the percentage of the predicted value achieved during the test.

Based on the medical evaluation and exercise test, individualized heart rate targets and training intensities were prescribed for each participant [22].

### 2.4. Functional and Clinical Assessments

Walking endurance was assessed using the 2-Minute Walk Test (2MWT), during which participants were instructed to walk as far as possible along a straight walkway over a 2-min period. The total distance covered was recorded as a measure of walking capacity [23].

Functional mobility and short-distance walking performance were assessed using the Timed Up and Go (TUG) [24] and Timed 25-Foot Walk (T25FW) tests [25], respectively. The TUG evaluates functional mobility by measuring the time required to stand up from a chair, walk 3 m, turn around, return to the chair, and sit down. The T25FW assesses short-distance walking speed by recording the time required to walk 25 feet (7.62 m) as quickly and safely as possible [25]. During both assessments, a wearable inertial measurement unit (Baiobit, BTS Bioengineering, Milan, Italy) was positioned on the participant to measure spatiotemporal gait parameters (speed and cadence) in addition to task completion time.

The Modified Fatigue Impact Scale (MFIS) is a 21-item self-report questionnaire specifically developed to assess the impact of fatigue in PwMS [26]. The instrument comprises three domains evaluating the physical (9 items), cognitive (10 items), and psychosocial (2 items) dimensions of fatigue. Each item is rated on a 5-point Likert scale ranging from 0 (“never”) to 4 (“almost always”), yielding a total score between 0 and 84. Higher scores indicate a greater perceived impact of fatigue on daily functioning [26].

Additional functional, balance, gait, and habitual physical activity measures reported in the Supplementary Figures S1 and S2 are described in the Supplementary Methods.

### 2.5. Exercise Intervention

Participants were scheduled to complete 24 supervised exercise sessions over 12 weeks, consisting of two 60-min sessions per week.

Sessions were conducted at the SUISM—School of Exercise and Sport Sciences, University of Torino, and were supervised by qualified exercise physiologists. The programme included HIIT on a cycle ergometer, resistance training, flexibility, and balance exercises. Exercise prescription was individualized according to baseline medical evaluation, exercise test results, functional status, symptoms, and perceived fatigue.

Participants were also encouraged to perform additional aerobic exercise at home to help achieve the 150 min per week of physical activity recommended by international guidelines and the World Health Organization [7,15]. However, this home-based activity was not structured, supervised, or consistently performed, and was therefore not considered part of the delivered intervention.

The aerobic component consisted of high-intensity interval training on a stationary cycle ergometer. Training workload was set as a percentage of the peak workload achieved during the baseline exercise test and individually adjusted to elicit the prescribed heart-rate response. Each session included a 5-min warm-up and a 5-min cool-down performed at low intensity.

The interval protocol was progressively increased over the 12-week programme as follows [Figure 2]:

- Weeks 1–2: six 1-min intervals at 75% heart rate reserve, interspersed with 1-min active recovery at 40% heart rate reserve.
- Weeks 3–4: eight intervals at 75% heart rate reserve, with active recovery at 40% heart rate reserve.
- Weeks 5–6: ten intervals at 75% heart rate reserve, with active recovery at 40% heart rate reserve.
- Weeks 7–9: ten intervals at 80% heart rate reserve, with active recovery at 40% heart rate reserve.
- Weeks 10–12: ten intervals at 85% heart rate reserve, with active recovery at 40% heart rate reserve.

Recovery between intervals consisted of light active pedalling. Heart rate, rating of perceived exertion using the Borg scale, symptoms, and power output were monitored during sessions. Exercise intensity could be adjusted when necessary, according to clinical status, fatigue, tolerance, and safety considerations.

Resistance training targeted the main upper- and lower-body muscle groups at a moderate intensity corresponding to approximately 40–60% of the indirectly estimated one-repetition maximum (1RM). Exercises included sit-to-stand variations, knee extension and flexion, seated row, pushing and pulling exercises, upper-limb strengthening, core-stability exercises, and functional lower-limb tasks. Based on each participant’s specific needs and functional capabilities, exercises were performed either using body weight alone or with additional resistance and specific equipment, including dumbbells (0.5–5 kg), ankle weights (1–2 kg), resistance bands, handles for pulling and exercises, and proprioceptive balance boards. Given the marked clinical and functional variability of MS, exercise selection, load, volume, range of motion, recovery periods, and progression were individualized according to each participant’s strength, distribution of motor deficits, balance, fatigue, thermosensitivity, and exercise tolerance. Load was progressively increased within the prescribed intensity range when exercises were completed with appropriate technique and without disproportionate symptoms.

Each session also included approximately 10 min of flexibility and balance exercises as complementary components. These exercises aimed to improve joint mobility, postural control, and movement confidence. Balance tasks were individually adapted and could include static and dynamic exercises, changes in base of support, weight shifting, and controlled functional movements.

Participants were supervised throughout each exercise session. Symptoms, perceived exertion, heart-rate response, fatigue, thermosensitivity, pain, motor control, balance, and general exercise tolerance were monitored. Exercise intensity or volume was reduced, recovery was extended, or an alternative exercise was selected when clinically indicated. The session or individual exercise could be discontinued if safe performance could not be maintained.

### 2.6. Unsupervised Follow-Up Period

After completion of the supervised exercise programme, participants entered an approximately three-month period during which no supervised exercise sessions or structured maintenance programme were provided by the study team. Participants were not instructed to discontinue physical activity and remained free to exercise independently. Habitual physical activity was assessed using the IPAQ short form; however, specific information regarding independently performed exercise, physiotherapy, or participation in programmes delivered at other facilities was not systematically recorded. The assessment performed before a subsequent exercise programme was used as the follow-up evaluation, before the new supervised programme commenced.

### 2.7. Adherence and Subgroup Definitions

Adherence was calculated as the percentage of supervised sessions attended out of the total number of scheduled sessions. Participants were classified as having high or low adherence according to the predefined adherence rate used in the analysis (cut off 75% of completed sessions) [27]. Given the observational nature of adherence and the small size of the low-adherence subgroup, analyses based on adherence were considered exploratory.

Baseline disability was stratified according to EDSS score using a cut-off of 4. Participants were categorized as having an EDSS score ≤ 4 or >4 [28]. This threshold was used to explore whether baseline disability was associated with different longitudinal trajectories in functional and exercise-related outcomes.

Additional exploratory models included EDSS category, adherence category, and their interaction with time to examine whether longitudinal trajectories differed according to baseline disability or adherence. Given the small sample size, the number of outcomes assessed, and subgroup imbalance, these analyses were considered exploratory and hypothesis-generating. No adjustment for multiple comparisons was applied.

### 2.8. Sample Size and Statistical Analysis

The sample size was determined by the number of participants from the available exercise programmes who met the eligibility criteria for the present analysis and had assessments available at the three relevant timepoints. Because this was a secondary analysis of an existing programme dataset rather than a prospectively planned confirmatory trial, no formal a priori sample-size calculation was performed.

Continuous variables were summarized as median and interquartile range (IQR), while categorical variables were described using absolute frequencies and percentages. Medians and IQRs were used as distribution-robust descriptive summaries.

Longitudinal changes were estimated using linear mixed-effects models, which provided model-based mean differences and 95% confidence intervals. The models reported in results section included timepoint as a categorical fixed effect, with T1 as the reference, and a participant-specific random intercept to account for repeated observations. These models were unadjusted. Paired Wilcoxon signed-rank tests were used as complementary non-parametric comparisons between individual timepoints. When the results of the two analytical approaches differed, interpretation was primarily based on the mixed-effects model estimates and their confidence intervals.

EDSS and adherence were examined in separate exploratory models and were not included simultaneously. All participants had complete observations at the three assessment timepoints. Given the limited sample size, no observations or participants were excluded solely on statistical grounds, and all participants included in the analytical cohort were retained in the analyses. Model assumptions were assessed by visual inspection of residual-versus-fitted and normal Q–Q plots.

Given the exploratory nature of the study and the absence of adjustment for multiplicity, *p*-values were interpreted as continuous measures rather than according to a dichotomous significance threshold. Interpretation focused primarily on effect estimates and their 95% confidence intervals.

Statistical analyses were performed using R version 4.6.0 [29], with the package “lme4” version 2.0–6 for mixed-effects models.

## 3. Results

### 3.1. Study Sample

The analytical cohort comprised 20 unique PwMS with complete data at the three required assessment timepoints, corresponding to 60 participant-time observations. The cohort was predominantly female (70%), with a median age of 53 years [IQR 44–60]. Median baseline EDSS was 4.0 [2.0–5.0]; twelve participants had an EDSS score ≤ 4, whereas eight participants had an EDSS score > 4. Most patients (*n* = 18; 90.0%) had relapsing-remitting MS. Median adherence to the exercise programme was 77.0% [64.0–84.3], with 14 participants classified as having high adherence and 6 as having low adherence, as shown in Table 1.

### 3.2. Exercise Testing and Estimated Cardiorespiratory Fitness

Numerical changes were observed in exercise-test-derived variables from T1 to T2, with smaller differences from baseline at FU. The percentage of maximal heart rate achieved was 81.0% [74.0–85.3] at T1, 81.0% [73.8–87.0] at T2, and 81.5% [75.5–89.5] at FU, with *p*-values of 0.999 for T1-T2, 0.306 for T2-FU.

Achieved workload varied from 93.0 W [76.5–113] at T1 to 100 W [84.5–120] at T2, and remained 100 W [71.3–123] at FU; *p*-values were 0.055 for T1–T2, 0.334 for T2–FU, and 0.320 for T1–FU. VO_2_ estimated using the FRIEND equation spanned from 18.4 mL/kg/min [15.9–22.9] at T1 to 20.2 mL/kg/min [18.1–22.1] at T2 and was 19.6 mL/kg/min [17.1–21.8] at FU; *p*-values were 0.080 for T1–T2, 0.224 for T2–FU, and 0.317 for T1–FU. Similarly, the percentage of predicted VO_2_ achieved varied from 69.5% [61.5–82.1] at T1 to 79.3% [72.1–81.9] at T2 and was 75.1% [64.7–82.7] at FU; *p*-values were 0.080 for T1–T2, 0.341 for T2–FU, and 0.268 for T1–FU. All confidence intervals included no change and were also compatible with modest positive differences. Although the magnitude of change was modest, the direction of change was coherent across workload, VO_2_ estimated using the FRIEND equation, and percentage of predicted VO_2_ achieved, with no clear deterioration after the unsupervised follow-up period.

### 3.3. Walking-Related Outcomes

Walking-related outcomes are shown in Table 2. The 2-min walk test increased from 121 m [103–138] at T1 to 126 m [111–141] at T2 and was 125 m [111–136] at FU. Although the paired T1–T2 comparison yielded *p* = 0.046, the main mixed-effects estimate was 5.00 m (95% CI −0.29 to 10.29). At follow-up, the estimate relative to T1 was 3.53 m (95% CI −1.76 to 8.82). These intervals indicate uncertainty compatible with no change as well as with modest positive differences.

TUG values were 11.00 s [9.11–13.70] at T1, 10.90 s [9.45–13.80] at T2, and 9.90 s [8.38–12.20] at FU. The paired T2–FU comparison yielded *p* = 0.012 for a 1.0 s lower median value; however, the main mixed-effects estimate for FU versus T1 was −0.81 s (95% CI −1.62 to 0.01), indicating compatibility with both no change and a small reduction in completion time. The corresponding T2-versus-T1 estimate was 0.26 s (95% CI −0.55 to 1.07). T25FW values were 7.54 s [5.85–7.98] at T1, 6.88 s [5.35–8.27] at T2, and 6.96 s [6.34–8.25] at FU. Mixed-model estimates were −0.11 s at T2 (95% CI −0.90 to 0.68) and −0.01 s at follow-up (95% CI −0.80 to 0.78).

Walking speed was 0.900 m/s [0.713–1.15] at T1, 0.885 m/s [0.745–1.32] at T2, and 1.06 m/s [0.888–1.25] at follow-up; *p*-values were 0.267, 0.911, and 0.135. Cadence was 106 steps/min [92.3–125] at T1, 104 [101–121] at T2, and 110 [95.0–118] at FU, with *p*-values of 0.341, 0.955, and 0.614.

### 3.4. Fatigue

Fatigue scores showed modest numerical fluctuations across the study period. The MFIS total score decreased from 38.0 [26.8–46.0] at T1 to 32.0 [23.5–38.0] at T2 and was 34.5 [25.8–46.3] at FU. Pairwise *p*-values were 0.331 for T1–T2, 0.513 for T2–FU, and 0.747 for T1–FU. In the main mixed-effects model, the estimates were −2.95 at T2 (95% CI −8.43 to 2.53) and −0.20 at follow-up (95% CI −5.68 to 5.28).

### 3.5. Linear Mixed-Effects Models

Linear mixed-effects models were used to estimate longitudinal changes while accounting for repeated measures (Table 3 and Figure 3).

Predicted VO_2_ remained unchanged at T2 (estimate 0.00 mL/kg/min, 95% CI −0.26 to 0.26; *p* = 0.974) and showed a small decrease at FU (estimate −0.34 mL/kg/min, 95% CI −0.60 to −0.08; *p* = 0.012). As predicted VO_2_ is a reference value calculated from demographic and anthropometric variables rather than from achieved exercise performance, this finding does not indicate a reduction in exercise capacity.

For MFIS, estimates were −2.95 at T2 (95% CI −8.43 to 2.53; *p* = 0.285) and −0.20 at follow-up (95% CI −5.68 to 5.28; *p* = 0.942).

When the EDSS category was included in the models (Figure 4), baseline disability was associated with the absolute level of several outcomes. For VO_2_ estimated using the FRIEND equation, EDSS ≤ 4 was associated with higher values compared with EDSS > 4 (estimate 5.9 mL/kg/min, 95% CI 0.51 to 11.33; *p* = 0.032). A similar direction was observed for percentage of predicted VO_2_ achieved (estimate 15.4 percentage points, 95% CI −0.91 to 31.79; *p* = 0.064) and workload (estimate 21.3 W, 95% CI −5.10 to 47.62; *p* = 0.112). For the 2-min walk test, the EDSS ≤ 4 estimate was 18 m (95% CI −8.53 to 45.40; *p* = 0.176), while for TUG it was −1.89 s (95% CI −5.77 to 1.98; *p* = 0.331) and for the T25FW −0.84 s (95% CI −2.23 to 0.54; *p* = 0.228).

EDSS-by-time interaction terms were not consistently observed across outcomes. In the interaction model, the 2-min walk test showed positive time estimates in the EDSS > 4 reference group at T2 and FU (T2 estimate 10.00 m, *p* = 0.018; follow-up estimate 8.59 m, *p* = 0.041), with negative EDSS ≤ 4 interaction terms (T2 interaction *p* = 0.122; FU interaction *p* = 0.117). For TUG, the FU estimate in the EDSS > 4 reference group was −1.92 s (95% CI −3.15 to −0.70), with an EDSS ≤ 4 × FU interaction estimate of 1.86 s (95% CI 0.28 to 3.45). For VO_2_ estimated using the FRIEND equation, EDSS ≤ 4 remained associated with higher absolute values in the interaction model (estimate 6.27 mL/kg/min; *p* = 0.032), while EDSS × time interactions were not clearly evident (T2 *p* = 0.794; FU *p* = 0.363). Thus, EDSS appeared to be more strongly related to baseline functional and cardiorespiratory status than to a uniform differential response to the exercise programme.

### 3.6. Exploratory Analyses by Adherence

Analyses stratified by adherence were considered exploratory. The low-adherence subgroup was small and appeared functionally different at baseline, with better values for several outcomes, including TUG, T25FW, 2-min walk test, walking speed, and VO_2_ estimated using the FRIEND equation. For this reason, adherence was not interpreted as a pure measure of exercise dose-response.

Despite these limitations, adherence-stratified trajectories suggested a possible difference in fatigue over time. In the high-adherence group, in the mixed-effects model including adherence, the low-adherence main effect for MFIS was 8.3 (95% CI −7.88 to 24.42; *p* = 0.309), while the low-adherence-by-time interactions were 9.0 at T2 (95% CI −2.31 to 20.26; *p* = 0.116) and 14.6 at FU (95% CI 3.29 to 25.86; *p* = 0.012). MFIS decreased from 38.0 [28.5–45.0] at T1 to 32.0 [22.5–35.5] at T2 and 30.0 [25.3–39.0] at FU. In the low-adherence group, MFIS increased from 36.5 [13.5–52.0] at T1 to 41.0 [26.8–50.8] at T2 and 50.5 [46.8–52.0] at FU.

For other adherence-stratified mixed models, interaction terms did not suggest a consistent dose-response pattern.

### 3.7. Summary of Longitudinal Pattern

Across the three assessment timepoints, modest numerical changes were observed in selected walking and exercise-testing outcomes, whereas most measures remained broadly stable. Most confidence intervals from the main models included no change, and the paired and mixed-model analyses were not always concordant. Because this was a small uncontrolled study, these trajectories cannot be attributed to the supervised exercise programme and should be interpreted as exploratory within-group observations that may inform the design and outcome selection of future controlled studies.

### 3.8. Safety

No adverse events occurred during the supervised exercise programme among the participants included in the analysis. No falls, exercise-related injuries, clinically relevant cardiovascular complications, or episodes of exercise intolerance requiring permanent programme discontinuation were observed.

## 4. Discussion

In this uncontrolled longitudinal study, selected functional and exercise-test-derived outcomes showed modest numerical changes after a 12-week supervised exercise programme, whereas most outcomes remained broadly stable during the subsequent three-month period without supervised training. The clearest numerical change concerned walking endurance, although the evidence was not consistent across the paired and mixed-model analyses. Short-distance walking performance, gait parameters, fatigue, and most exercise-test-derived outcomes did not show statistically robust changes. Given the absence of a control group, these findings describe within-group trajectories over time and should not be interpreted as evidence that the programme caused improvement or prevented functional decline.

Walking endurance, assessed by the 2-min walk test, showed the clearest functional signal after the supervised phase and remained above baseline at follow-up. Exercise-test-derived outcomes, including achieved workload, VO_2_ estimated using the FRIEND equation, and percentage of predicted VO_2_ achieved, changed in a concordant direction after training and were partially preserved after the interruption of supervised sessions. By contrast, short-distance walking performance, gait parameters, fatigue scores, showed more heterogeneous and generally non-significant trajectories in this cohort of PwMS. Overall, these findings describe heterogeneous within-group patterns across outcomes; they do not demonstrate that the supervised exercise programme supported functional performance, maintained functional reserve, or prevented functional decline.

The median distance covered during the 2MWT increased by 5 m from T1 to T2. Although the paired comparison yielded a *p*-value of 0.046, the corresponding mixed-model estimate was 5.00 m, with a 95% confidence interval including no change (95% CI −0.29 to 10.29; *p* = 0.063). The statistical evidence should therefore be considered inconsistent across analytical approaches. Moreover, although the 2MWT has demonstrated good reliability and validity in PwMS, a well-established anchor-based threshold for clinically meaningful change in the clinician-administered 2MWT has not been consistently defined for heterogeneous MS populations [23]. Consequently, the observed 5-m change should be interpreted as small and of uncertain clinical relevance rather than as evidence of a clinically meaningful improvement. The individual trajectory further indicates substantial heterogeneity in response.

In contrast, the Timed 25-Foot Walk did not show a consistent model-based change despite a favourable numerical difference after the supervised programme. This distinction may indicate that, in this cohort, a short-distance walking-speed test was less responsive to change than an endurance-oriented walking test.

The Timed 25-Foot Walk captures rapid short-distance performance and may be influenced by day-to-day variability, compensatory strategies, baseline disability, or ceiling effects, whereas the 2-min walk test may better reflect integrated walking endurance and functional reserve. This interpretation is supported by previous evidence demonstrating that the 2-min walk test reflects sustained ambulatory performance and captures physiological responses that differ from those assessed by shorter walking tests [30,31]. Compared with brief speed-based measures, endurance-oriented walking tests integrate aerobic capacity, fatigue resistance and walking efficiency, making them particularly suitable for detecting functional adaptations following exercise interventions in PwMS [30,31].

The TUG remained broadly stable across the observation period, with a favourable descriptive trajectory at follow-up. Given the small sample size and the absence of a non-exercising control group, this pattern should not be interpreted as definitive evidence of delayed improvement. However, the approximately 1 s difference observed between T2 and FU cannot be assumed to represent a clinically meaningful change. Considering the non-significant T1–FU comparison and the uncontrolled study design, the TUG findings are best interpreted as showing descriptive stability and individual variability over time.

The TUG reflects not only walking speed, but also sit-to-stand ability, turning, balance, coordination, and confidence during movement. These components may respond differently from pure walking endurance and may be influenced by learning effects, spontaneous variability, or changes in daily motor behaviour after the supervised phase. Therefore, the TUG findings indicate that dynamic mobility remained broadly stable during the follow-up period, without evidence of a clinically meaningful improvement.

A second relevant observation was the concordant numerical direction of the exercise-test-derived outcomes. Achieved workload, VO_2_ estimated using the FRIEND equation, and percentage of predicted VO_2_ achieved were numerically higher after the supervised programme and remained partially above baseline at FU. However, the corresponding mixed-model estimates were imprecise, and all 95% confidence intervals included no change. Moreover, because these variables were obtained from the same exercise test and are mathematically related, their concordance does not represent independent corroboration. The observed pattern is therefore hypothesis-generating and does not establish an intervention-related change in exercise tolerance or performance-derived cardiorespiratory capacity. Exercise tolerance is particularly relevant in PwMS, who often present reduced cardiorespiratory fitness, deconditioning, and limited exercise capacity [32]. Even small improvements in exercise-test performance may reflect a greater ability to sustain external work and may contribute to functional reserve, especially when combined with preserved walking performance.

An important descriptive observation was that most functional measures remained broadly stable during the approximately three-month period without supervised training. In PwMS, the longitudinal trajectory of functional outcomes is clinically relevant; however, in the present uncontrolled cohort, the absence of observed decline cannot be interpreted as preservation attributable to the programme. Natural variability, repeated-testing effects, changes in clinical status, and physical activity performed outside the supervised sessions cannot be separated from any possible programme effect.

Current recommendations recognize regular aerobic and resistance exercise as components of MS management [32]. Nevertheless, the present findings do not establish that the supervised programme was responsible for the subsequent stability of functional outcomes. Controlled studies are required to determine whether repeated supervised programmes, booster sessions, hybrid models, or structured home-based programmes influence longer-term trajectories.

Future studies should specifically test whether repeated supervised programmes, booster sessions, hybrid models, or structured home-based maintenance programmes can prolong or amplify these effects.

Fatigue showed a favourable but inconclusive trajectory. MFIS total score decreased numerically after the supervised programme, with partial attenuation at FU. This is not unexpected, as fatigue in MS is multidimensional and may be influenced by neurological impairment, sleep, mood, thermosensitivity, physical deconditioning, medication, and behavioural factors [12,13]. The exploratory adherence analysis suggested a more favourable MFIS trajectory among participants with high adherence, while fatigue worsened in the low-adherence subgroup. This finding is hypothesis-generating and should be interpreted cautiously because adherence was not randomized, the low-adherence group was small, and baseline functional differences were present. The observed association should not be interpreted as evidence of a causal dose–response relationship. Adherence may reflect baseline health status, motivation, symptom burden, or other unmeasured clinical and behavioural factors in addition to the amount of exercise performed. Future studies should assess adherence as a multidimensional construct, including attendance, exercise dose, intensity achieved, perceived effort, symptom fluctuations, barriers, motivation, and physical activity performed outside supervised sessions.

Baseline disability, assessed using EDSS, was associated with absolute performance levels, particularly for cardiorespiratory and walking-related outcomes. Participants with lower disability generally showed better functional and exercise-test-derived performance, consistent with greater baseline functional reserve. However, EDSS-by-time interactions did not indicate a uniform differential response to the exercise programme. Given the small subgroups and the inconsistent interaction terms, the apparent differences in longitudinal trajectories may reflect baseline performance, individual variability, or chance. Therefore, these analyses do not provide evidence of a differential response to the exercise programme according to baseline EDSS. These observations support the need for disability-stratified analyses in future trials and for outcome selection tailored to baseline functional level.

Habitual physical activity did not show a sustained increase. This distinction is important from an implementation perspective. Changes in exercise-test-derived capacity or supervised-session performance do not automatically translate into greater spontaneous physical activity in daily life. In PwMS, barriers such as fatigue, transportation, fear of symptom worsening, lack of confidence, environmental constraints, and fluctuating symptoms may limit the transition from supervised exercise to autonomous activity. Therefore, supervised training and behavioural maintenance should be considered complementary targets.

This apparent discrepancy between numerical changes in exercise-test performance and unchanged habitual physical activity has been reported consistently in MS studies. The physical activity performed in daily life is strongly influenced by behavioural factors, including self-efficacy in relation to exercise, perception of symptoms, and confidence in managing disease-related barriers. Therefore, changes in physical capacity alone may not be enough to encourage long-term changes in everyday physical activity, unless behavioural strategies are incorporated into exercise programmes [33,34].

Future programmes may need to combine exercise prescription with behavioural support, self-monitoring tools, goal setting, education, and strategies to overcome individual barriers to daily physical activity.

Taken together, these results describe selective numerical changes and broad stability rather than generalized functional change. They should not be interpreted as evidence of clinically meaningful improvement, prevention of deconditioning, or maintenance attributable to the programme. Walking endurance and exercise-test-derived variables may represent candidate endpoints for future controlled studies because they showed larger numerical changes than several other measures, but their responsiveness requires confirmation.

This study has several strengths. First, the longitudinal design included assessments before and after the supervised exercise programme and after a subsequent period without supervised training, allowing the trajectory of functional change to be described beyond the immediate post-intervention phase. Second, the intervention was individualized and medically supervised, with exercise intensity derived from baseline exercise testing and adapted to clinical status, symptoms, and tolerance. Third, the outcome battery was multidimensional, including walking performance, functional mobility, fatigue, balance and gait parameters, and exercise-test-derived capacity. Finally, the use of mixed-effects models allowed repeated measures to be analysed within a longitudinal framework and enabled exploratory evaluation of disability and adherence patterns.

Several limitations should also be acknowledged. The sample size was small, and the study did not include a non-exercising control group; therefore, causal inference is limited and spontaneous variability, learning effects, regression to the mean, or changes in disease-related symptoms cannot be excluded. The exploratory analyses by EDSS and adherence were based on small and imbalanced subgroups and should be considered hypothesis-generating.

The principal limitation of this study is the absence of a control group. Consequently, the observed within-group trajectories cannot be causally attributed to the supervised exercise programme. Natural variability in MS symptoms, repeated-testing or learning effects, regression to the mean, changes in clinical status or treatment, and physical activity performed outside the supervised sessions may have influenced the results. Moreover, the absence of statistically significant deterioration during follow-up cannot be interpreted as evidence that the programme preserved function or prevented decline. The small sample size further limits statistical precision and increases the uncertainty surrounding both the overall and subgroup estimates.

Adherence was observational rather than randomized and may reflect broader clinical, motivational, and behavioural factors in addition to exercise exposure. The follow-up period was limited to approximately three months, and longer observation is needed to characterize functional trajectories over time and determine whether any changes are attributable to supervised exercise. Finally, VO_2_ was estimated from cycle ergometer performance rather than directly measured by cardiopulmonary gas exchange analysis, which limits the precision of cardiorespiratory fitness assessment.

## 5. Conclusions

In this small uncontrolled cohort of PwMS, modest within-group changes were observed in selected walking and exercise-test-derived outcomes after a 12-week supervised exercise programme, whereas most measures remained broadly stable during the subsequent three-month period without supervised training. The findings were not consistently supported across statistical approaches and should be considered exploratory. Because of the absence of a control group, no causal effect or programme-related preservation of function can be inferred. Larger controlled studies are required to determine the effectiveness of the intervention and the trajectory of outcomes after supervision is withdrawn.

## Figures and Tables

**Figure 1:**
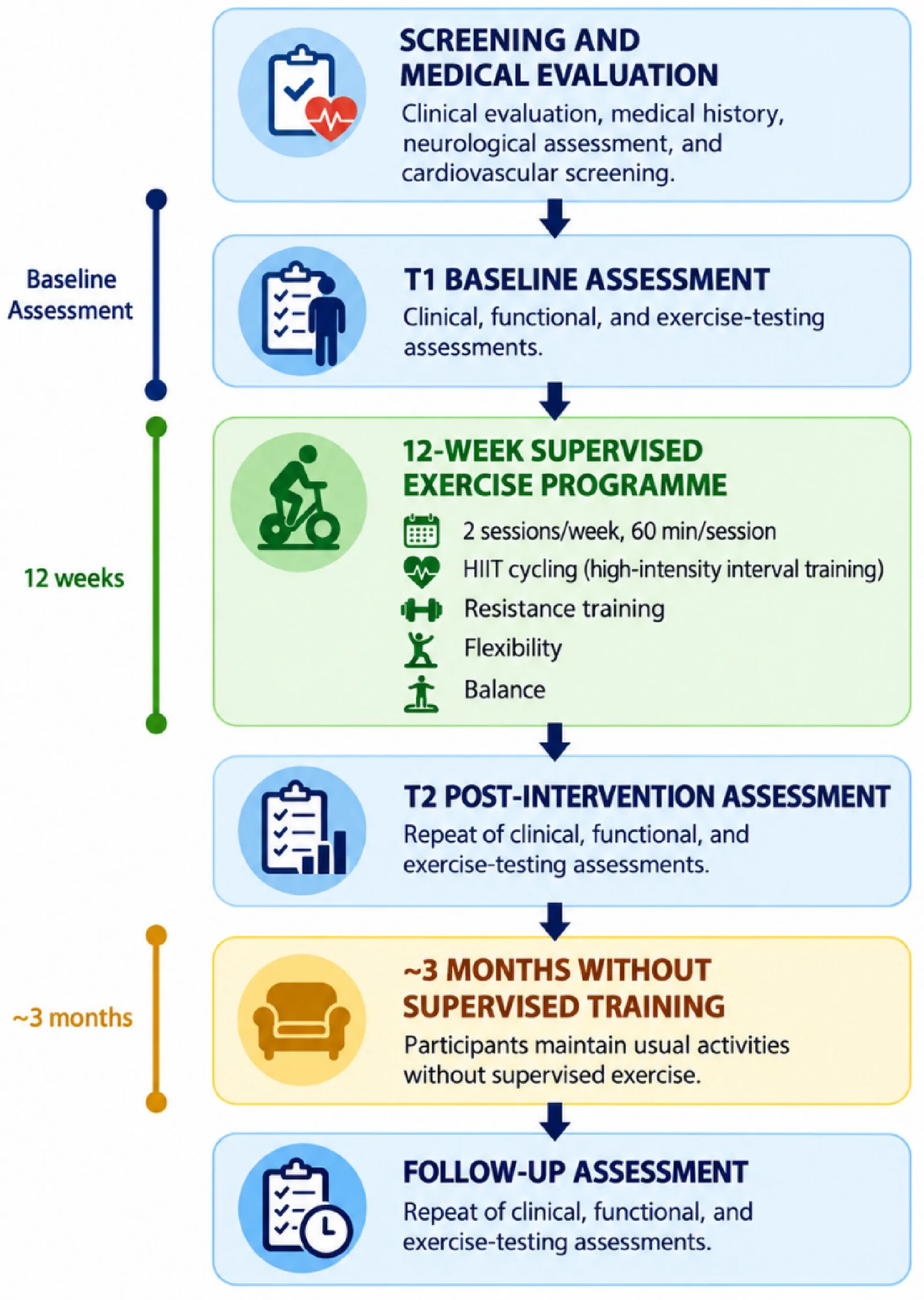
Study design and assessment timeline.

**Figure 2:**
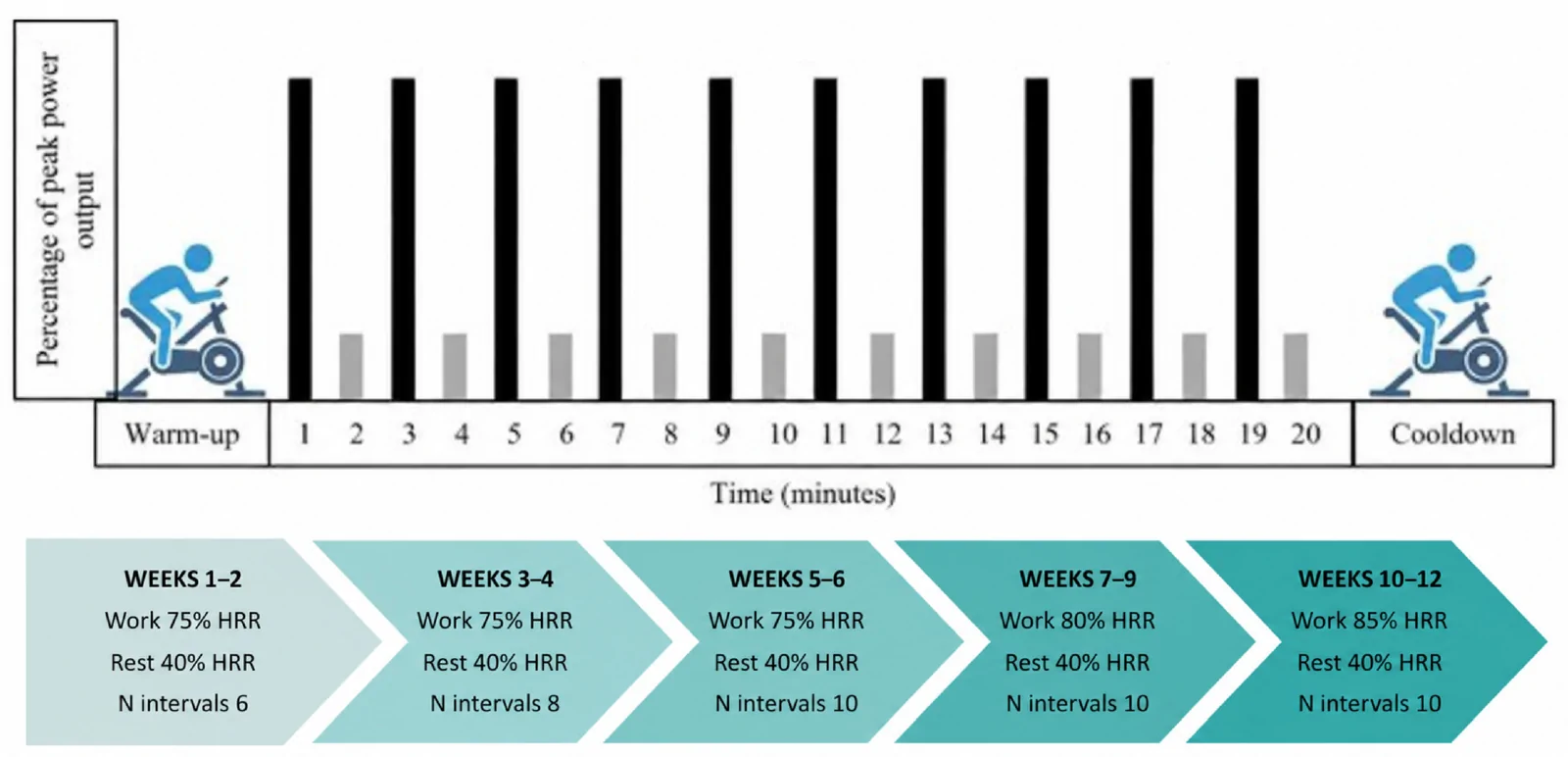
HIIT protocol.

**Figure 3:**
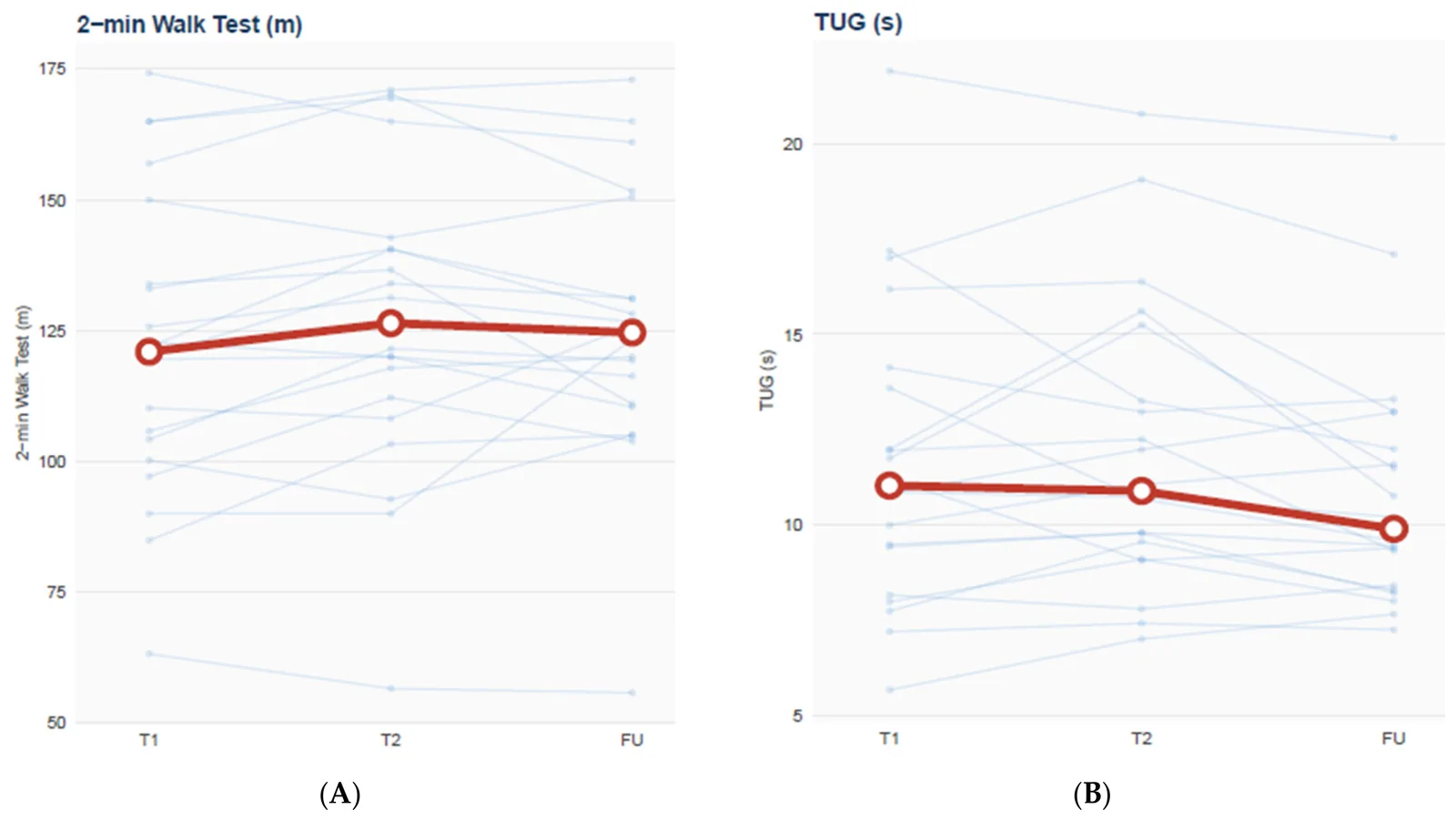

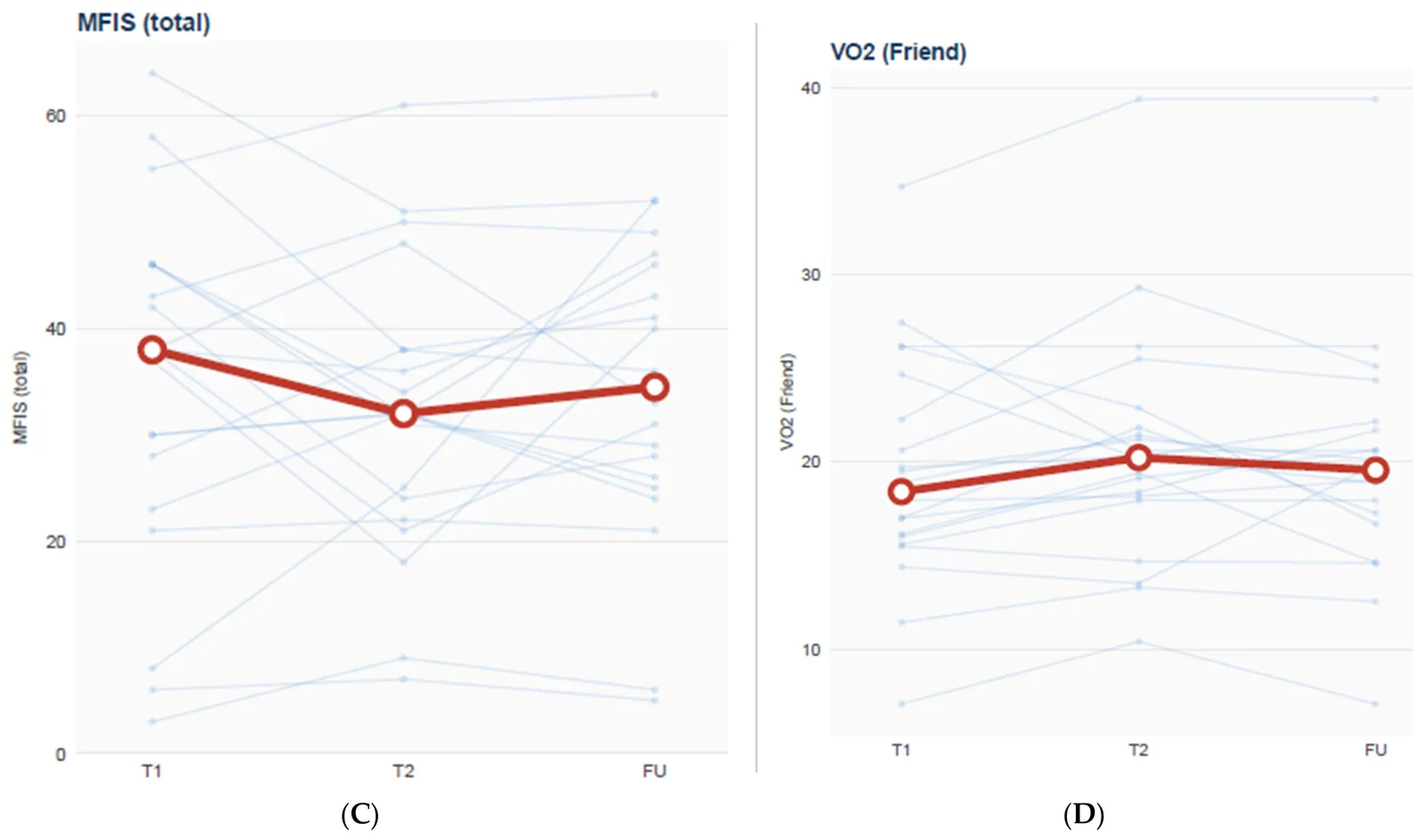
Individual trajectories of key outcomes. Individual longitudinal trajectories of selected functional and exercise-testing outcomes. Spaghetti plots showing individual changes from T1 to T2 and FU for: (**A**) 2-min walk test, (**B**) Timed Up and Go, (**C**) MFIS total score, and (**D**) VO_2_ estimated using the FRIEND equation. Thin lines represent individual participants; the thicker line represents the group-level trajectory. Lower values indicate better performance for Timed Up and Go and MFIS, whereas higher values indicate better performance for 2-min walk test and VO_2_ estimated using the FRIEND equation.

**Figure 4:**
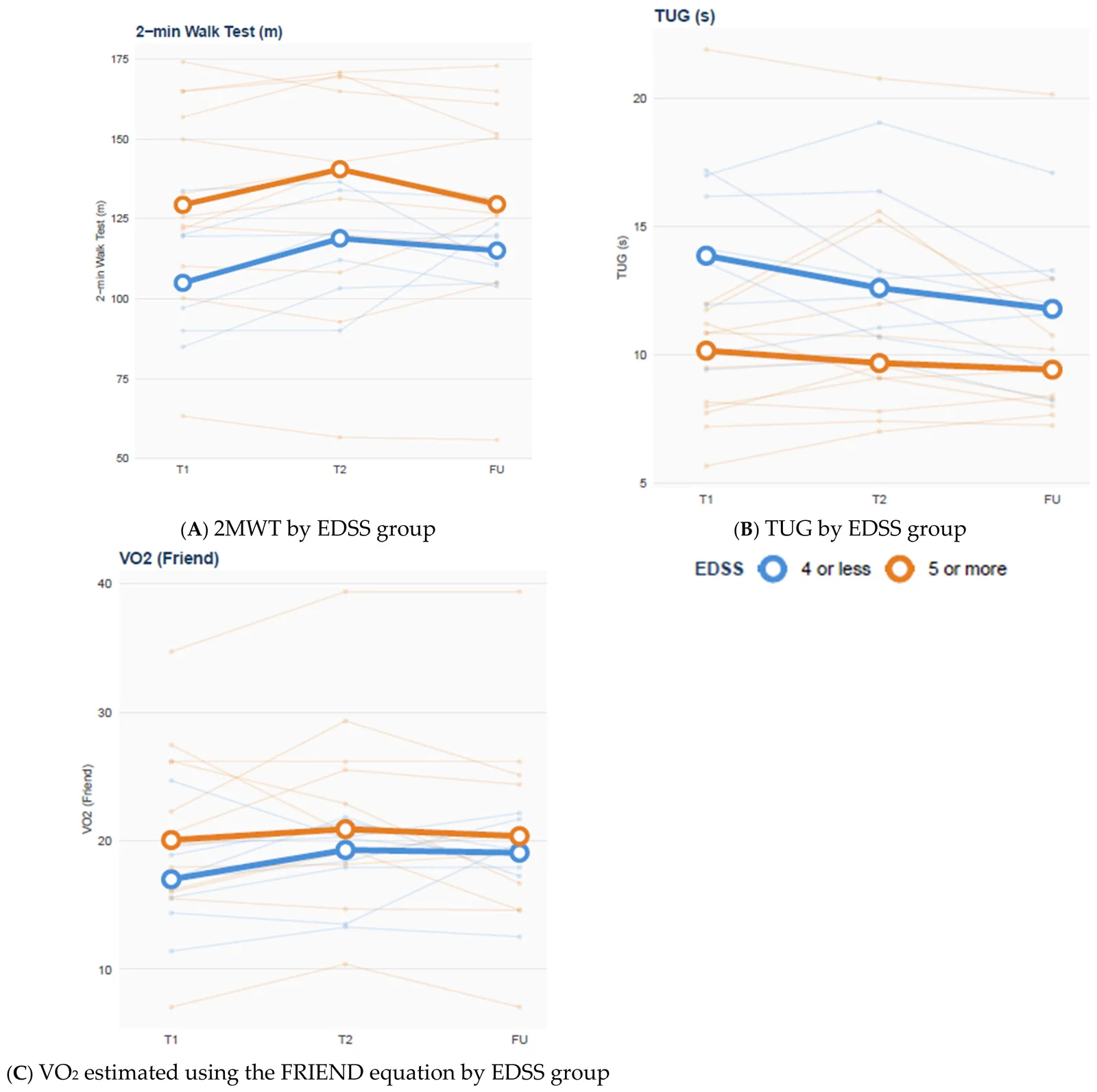
EDSS-stratified trajectories. Long. Longitudinal trajectories according to baseline disability. Changes in selected outcomes from T1 to T2 and FU stratified by EDSS category (EDSS ≤ 4 in blue vs. EDSS > 4 in orange). The figure illustrates differences in absolute performance levels and exploratory differences in longitudinal trajectories according to baseline disability.

**Table 1:** Baseline characteristics of the study population.

Variable	Overall Cohort, N = 20
Age, years	53 [44–60]
Sex, female	14/20, 70%
MS phenotype, relapsing-remitting	18/20, 90%
MS phenotype, primary progressive	2/20, 10%
EDSS	4 [2–5]
EDSS ≤ 4	12/20, 60%
EDSS > 4	8/20, 40%
Programme adherence, %	77 [64–84]
High adherence	14/20, 70%
Low adherence	6/20, 30%

Values are reported as median [IQR] or *n* (%). EDSS: Expanded Disability Status Scale; MS: Multiple Sclerosis. Overall longitudinal changes during the supervised programme and unsupervised follow-up.

**Table 2:** Longitudinal changes in functional performance, gait, fatigue, and exercise-testing outcomes. Values are median [IQR] unless otherwise indicated. *p*-values refer to paired comparisons between timepoints.

Outcome	T1	T2	Follow-Up	T1–T2 *p*	T2-FU *p*	T1-FU *p*
**Functional performance**						
TUG, s	11.00 [9.11–13.70]	10.90 [9.45–13.80]	9.90 [8.38–12.20]	0.422	0.012	0.126
T25FW, s	7.54 [5.85–7.98]	6.88 [5.35–8.27]	6.96 [6.34–8.25]	0.173	0.896	0.390
2-min walk test, m	121 [103–138]	126 [111–141]	125 [111–136]	0.046	0.225	0.235
**Gait parameters**						
Walking speed, m/s	0.900 [0.713–1.15]	0.885 [0.745–1.32]	1.06 [0.888–1.25]	0.267	0.911	0.135
Cadence, steps/min	106 [92.3–125]	104 [101–121]	110 [95.0–118]	0.341	0.955	0.614
**Fatigue**						
MFIS total	38.0 [26.8–46.0]	32.0 [23.5–38.0]	34.5 [25.8–46.3]	0.331	0.513	0.747
**Exercise-testing outcomes**						
Workload, W	93.0 [76.5–113.0]	100.0 [84.5–120.0]	100.0 [71.3–123.0]	0.055	0.334	0.320
VO_2_ estimated using the FRIEND equation, mL/kg/min	18.4 [15.9–22.9]	20.2 [18.1–22.1]	19.6 [17.1–21.8]	0.080	0.224	0.317
% predicted VO_2_ achieved	69.5 [61.5–82.1]	79.3 [72.1–81.9]	75.1 [64.7–82.7]	0.080	0.341	0.268

**Table 3:** Linear mixed-effects models for longitudinal changes in main outcomes. Estimates derive from linear mixed-effects models with time as categorical fixed effect and participant as random effect. T1 was the reference timepoint. Negative estimates indicate lower values compared with T1; for TUG and T25FW, negative values indicate faster performance.

Outcome	Contrast	Estimate	95% CI	*p*-Value
2-min walk test, m	T2 vs. T1	5.00	−0.29 to 10.29	0.063
2-min walk test, m	FU vs. T1	3.53	−1.76 to 8.82	0.187
TUG, s	T2 vs. T1	0.26	−0.55 to 1.07	0.520
TUG, s	FU vs. T1	−0.81	−1.62 to 0.01	0.051
T25FW, s	T2 vs. T1	−0.11	−0.90 to 0.68	0.787
T25FW, s	FU vs. T1	−0.01	−0.80 to 0.78	0.976
MFIS total	T2 vs. T1	−2.95	−8.43 to 2.53	0.285
MFIS total	FU vs. T1	−0.20	−5.68 to 5.28	0.942
Workload, W	T2 vs. T1	7.40	−1.78 to 16.58	0.112
Workload, W	FU vs. T1	4.65	−4.53 to 13.83	0.315
VO_2_ estimated using the FRIEND equation, mL/kg/min	T2 vs. T1	1.28	−0.28 to 2.83	0.106
VO_2_ estimated using the FRIEND equation, mL/kg/min	FU vs. T1	0.49	−1.07 to 2.04	0.532
% predicted VO_2_ achieved	T2 vs. T1	4.72	−0.95 to 10.40	0.101
% predicted VO_2_ achieved	FU vs. T1	2.45	−3.23 to 8.12	0.392
Predicted VO_2_, mL/kg/min	T2 vs. T1	0.00	−0.26 to 0.26	0.974
Predicted VO_2_, mL/kg/min	FU vs. T1	−0.34	−0.60 to −0.08	0.012

## Data Availability

The data supporting the findings of this study are available from the authors upon reasonable request. Requests should be addressed to Filippo Tarditi (f.tarditi@unito.it) or Luca Beratto (luca.beratto@unito.it). The data are not publicly available due to privacy and ethical restrictions.

## Supplementary Materials

The following supporting information can be downloaded at: https://www.mdpi.com/article/10.3390/life16101630/s1, Figure S1: Complete spaghetti plots for all outcomes; Figure S2: Complete EDSS and Adherence-stratified trajectories; Table S1: EDSS-stratified mixed-effects models: interaction terms; Table S2: Exploratory adherence mixed-effects models.

## Abbreviations

The following abbreviations are used in this manuscript:

2MWT	2-Minute Walk Test
CI	confidence interval
EDSS	Expanded Disability Status Scale
FRIEND	Fitness Registry and the Importance of Exercise National Database
FU	follow-up assessment
HIIT	high-intensity interval training
HRR	heart rate reserve
IQR	interquartile range
MET	metabolic equivalent of task
MFIS	Modified Fatigue Impact Scale
MS	multiple sclerosis
PwMS	people with multiple sclerosis
T1	baseline assessment
T2	post-intervention assessment
T25FW	Timed 25-Foot Walk
TUG	Timed Up and Go
VO_2_	oxygen uptake
VO_2_max	maximal oxygen uptake
VO_2_peak	peak oxygen uptake
