# Supplementary material for: Changes in Functional Performance During and After a Supervised Exercise Programme in People with Multiple Sclerosis: A Secondary Uncontrolled Longitudinal Study
Source: Life (Basel). 2026 Sep 29;16(10):1630. doi: 10.3390/life16101630 (PMC13706077; doi:10.3390/life16101630)
Supplement: Supplementary file 1 [file life-16-01630-s001.zip › life-4494706-supplementary.pdf]

life

MDPI

Article

Changes in Functional Performance During and After a Supervised Exercise Programme in People with Multiple Sclerosis: A Secondary Uncontrolled Longitudinal Study

Filippo Tarditi[1]*, Luca Beratto[1,2], Francesco Borrelli[1], Emanuele Koumantakis[3,4], Giulia Actis Dato[1], Gaetano Rossi[1], Lara Bressy[2], Elena Turiano[5], Alberto Rainoldi[1,2], Andrea Silvio Benso[1,6], Anna Mulasso[1,2]

[1] Department of Medical Sciences, University of Torino, Torino, Italy;
[2] NeuroMuscularFunction | Research group, School of Exercise and Sport Sciences, SUISM, University of Torino, Torino, Italy;
[3] Center for Biostatistics, Epidemiology, and Public Health, Department of Clinical and Biological Sciences, University of Torino, Italy
[4] Cancer Epidemiology Unit, Città della Salute e della Scienza di Torino University Hospital, Torino, Italy
[5] 160CM APS, Torino, Italy
[6] Division of Endocrinology, Diabetology and Metabolism, Department of Medical Sciences, University of Torino, Torino, Italy
* Correspondence: f.tarditi@unito.it

Supplementary Methods

Additional functional and physical activity assessments

Lower-limb functional performance was assessed using the Short Physical Performance Battery (SPPB), comprising standing balance, usual-pace walking speed over 4-m, and five repeated chair stands. Each component was scored from 0 to 4, providing a total score ranging from 0 to 12, with higher scores indicating better physical performance.

Upper-limb strength was assessed using a hand-grip dynamometer (CAMRY EH101, Sensun Weighing Apparatus Group Ltd, Guangdong, China). The test was performed with the participant in a seated position, with the elbow positioned with the arms alongside the body and the elbows fully extended. Three trials were performed for each hand, with 60-second rest interval between trials. The highest value obtained from both hands was retained for analysis and expressed in kilograms.

Static balance was assessed under eyes-open (OE) and eyes-closed (CE) conditions using a wearable inertial measurement unit (Baiobit, BTS Bioengineering, Italy). Participants maintained a standardized parallel standing position, with their feet hip-width apart and their arms relaxed alongside the body for 30 seconds, with the sensor positioned at the level of the S1 vertebrae . Data were acquired at 200 Hz and processed using dedicated software (Baiobit, BTS Bioengineering, Italy). The balance index represented (the relationship between the length of the trajectory and the area of the ellipse) provides an overall measure of postural system performance, with values deviating substantially from 1 indicating poorer postural control.

During the Timed Up and Go and Timed 25-Foot Walk tests, the wearable inertial measurement unit (Baiobit, BTS Bioengineering, Italy) was positioned using semi-elastic belts approximately at the L1 and S1 vertebral levels, respectively.

Data was acquired at 200 Hz and processed using dedicated software (Baiobit, BTS Bioengineering, Italy) to derive walking speed and cadence. Each test was performed two times, and the best performance was retained for analysis.

Habitual physical activity was assessed using the short form of the International Physical Activity Questionnaire (IPAQ-SF), referring to physical activity performed during the preceding seven days. Weekly energy expenditure was expressed as MET-minutes per week. Walking, moderate-

intensity activity, and vigorous-intensity activity were weighted using the standard coefficients of 3.3, 4.0, and 8.0 METs, respectively. Total MET-minutes per week were calculated by summing the MET-minutes obtained across the three activity-intensity categories.
These additional outcomes were included to provide a broader descriptive characterization of individual trajectories and were not used to support the principal interpretation of the longitudinal findings.

Supplementary Tables

Supplementary Table S1. EDSS-stratified mixed-effects models: interaction terms

Outcome	Contrast	Estimate	95% CI	p-value
2MWT, m	T2 vs T1 in EDSS >4	10.00	1.77 to 18.24	0.018
2MWT, m	FU vs T1 in EDSS >4	8.59	0.35 to 16.83	0.041
2MWT, m	EDSS ≤ 4 x T2	-8.34	-18.97 to 2.30	0.122
2MWT, m	EDSS ≤ 4 x FU	-8.44	-19.08 to 2.19	0.117
TUG, s	FU vs T1 in EDSS >4	-1.92	-3.15 to -0.70	0.003
TUG, s	EDSS ≤ 4 x FU	1.86	0.28 to 3.45	0.022
VO_2 estimated using the FRIEND equation, mL/kg/min	EDSS ≤ 4	6.27	0.55 to 11.99	0.032
VO_2 estimated using the FRIEND equation, mL/kg/min	EDSS ≤ 4 x T2	0.42	-2.78 to 3.62	0.794
VO_2 estimated using the FRIEND equation, mL/kg/min	EDSS ≤ 4 x FU	-1.46	-4.66 to 1.74	0.363

Footnote: Interaction models used EDSS >4 and T1 as reference categories. The EDSS ≤ 4 x time terms indicate the additional difference in longitudinal change among participants with EDSS ≤ 4 compared with EDSS >4.

Supplementary Table S2. Exploratory adherence mixed-effects models

Outcome	Contrast	Estimate	95% CI	p-value
MFIS total	T2 vs T1 in high adherence	-5.64	-11.82 to 0.54	0.073
MFIS total	FU vs T1 in high adherence	-4.57	-10.75 to 1.61	0.144
MFIS total	Low adherence	0.42	-17.01 to 17.85	0.962
MFIS total	Low adherence x T2	8.98	-2.31 to 20.26	0.116
MFIS total	Low adherence x FU	14.57	3.29 to 25.86	0.012
TUG, s	Low adherence x T2	1.03	-0.69 to 2.76	0.235
TUG, s	Low adherence x FU	1.76	0.04 to 3.49	0.046
2MWT, m	Low adherence x T2	1.26	-10.58 to 13.11	0.831
2MWT, m	Low adherence x FU	-1.66	-13.50 to 10.19	0.780
VO_2 estimated using the FRIEND equation	Low adherence x T2	1.45	-1.99 to 4.90	0.400
VO_2 estimated using the FRIEND equation	Low adherence x FU	-0.08	-3.52 to 3.37	0.964
Workload, W	Low adherence x T2	2.52	-17.93 to 22.98	0.805
Workload, W	Low adherence x FU	-5.45	-25.91 to 15.00	0.595

Footnote: High adherence and T1 were the reference categories. These analyses were considered exploratory because the low-adherence subgroup was small and functionally different at baseline.

Supplementary figures

Supplementary Figure S1. Complete spaghetti plots for all outcomes

Supplementary Figure 1. Individual trajectories for all measured outcomes. Spaghetti plots showing individual changes across T1, T2, and follow-up for functional performance, balance, gait, fatigue, habitual physical activity, and exercise-testing outcomes.

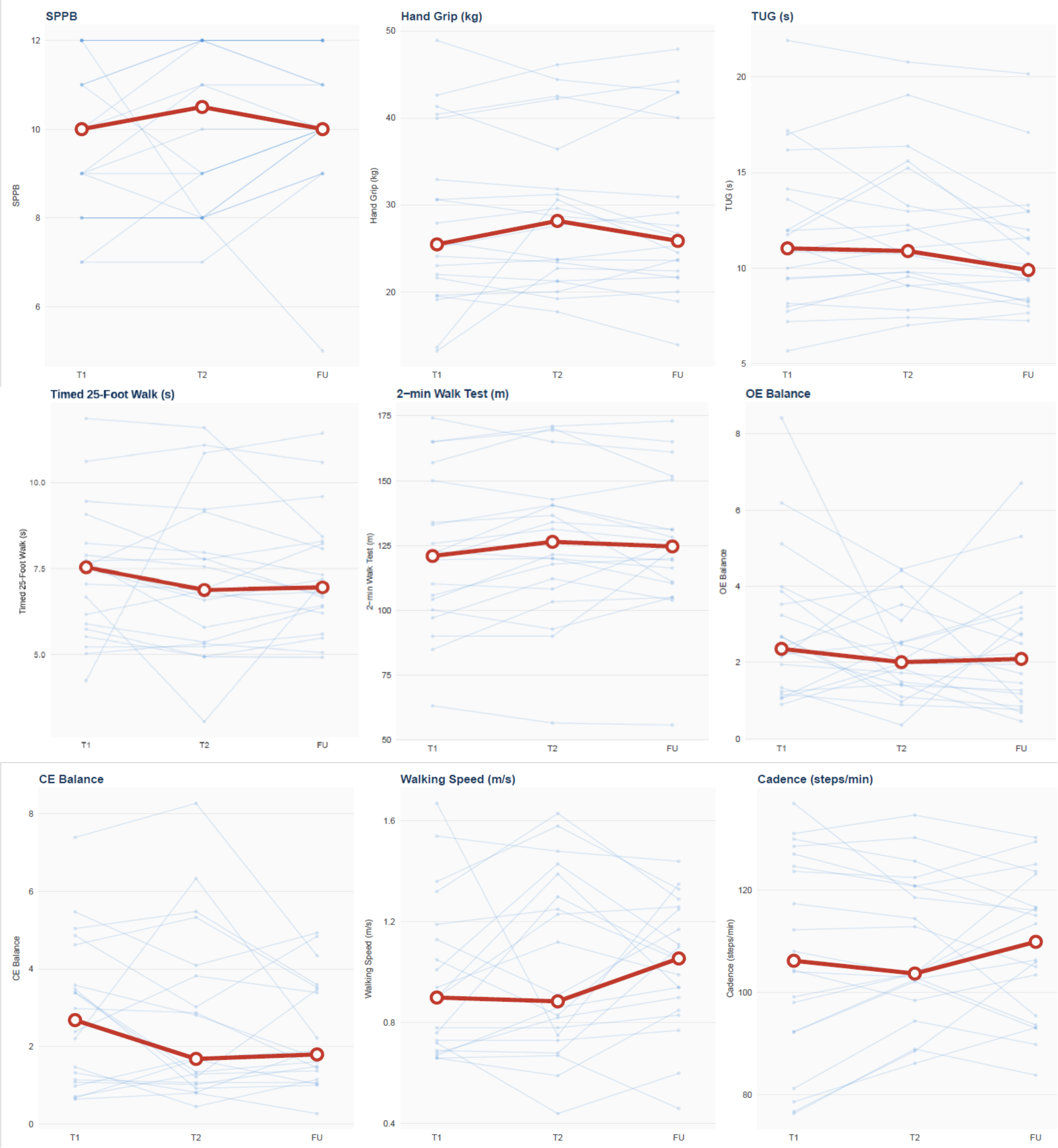

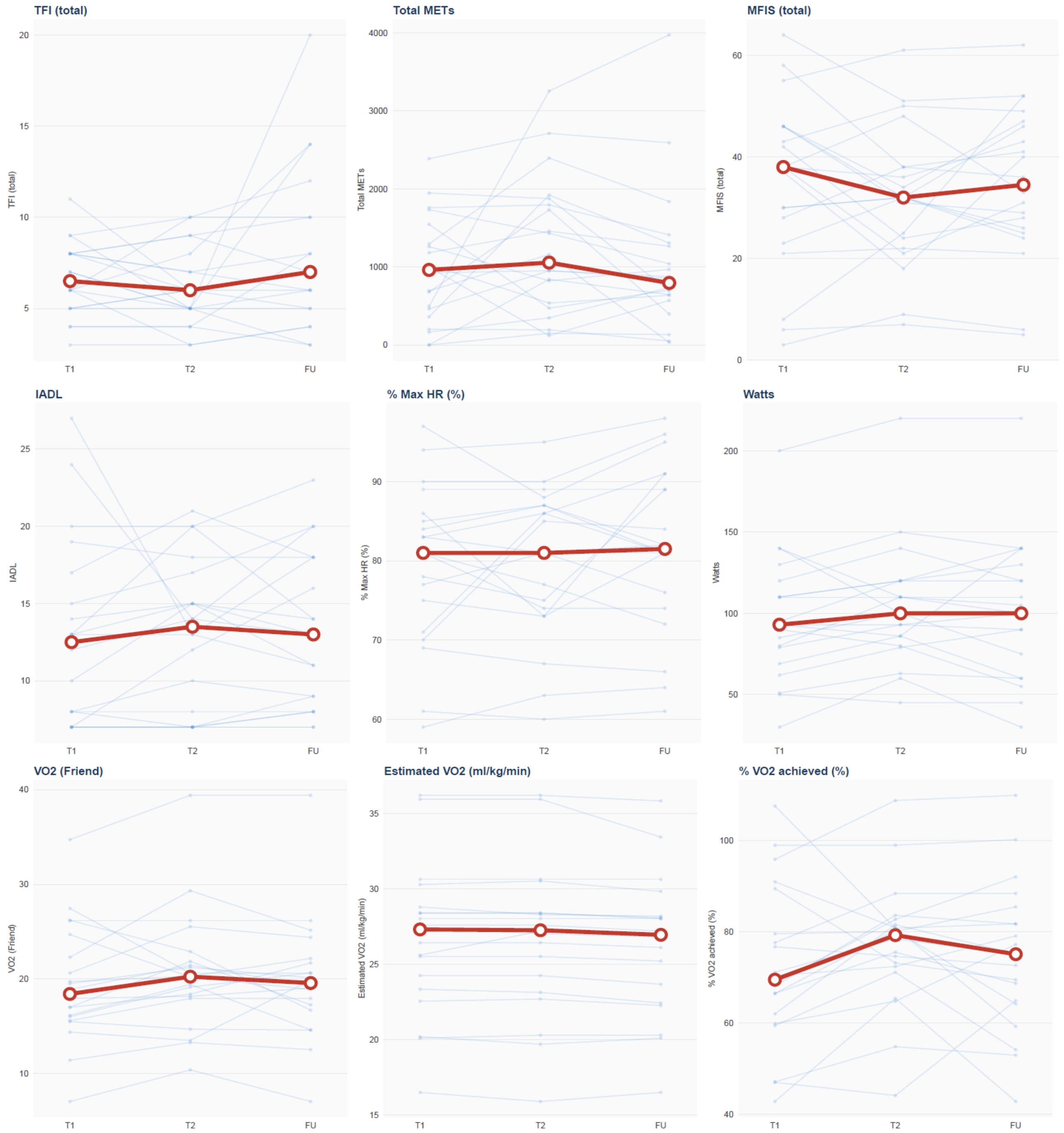

Supplementary Figure S2. Complete EDSS and Adherence-stratified trajectories

Supplementary Figure 2. Exploratory EDSS and adherence-stratified trajectories. Individual trajectories stratified by EDSS and adherence category. These analyses were considered exploratory because the low-adherence subgroup was small and functionally different at baseline.

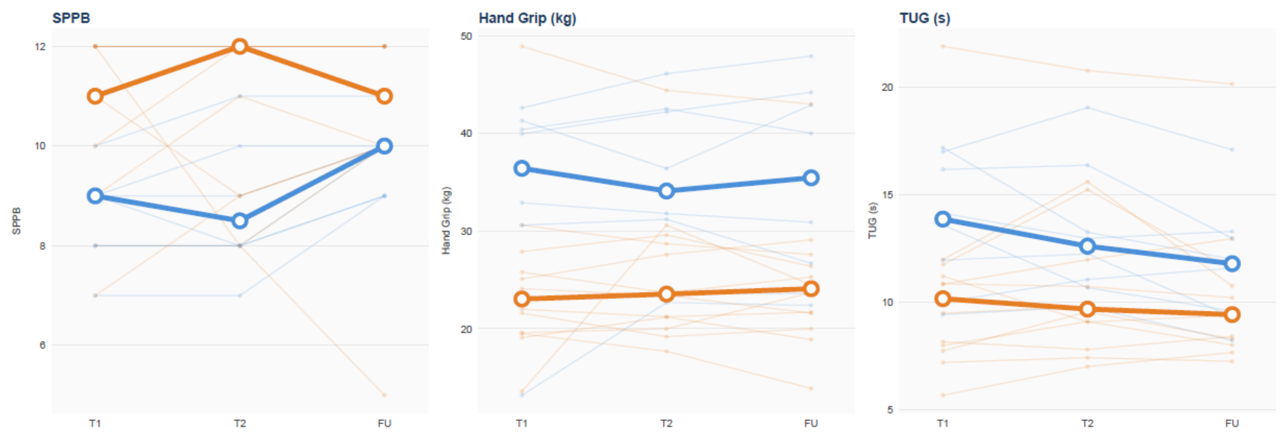
SPPB
Hand Grip (kg)
TUG (s)
T1
T2
FU

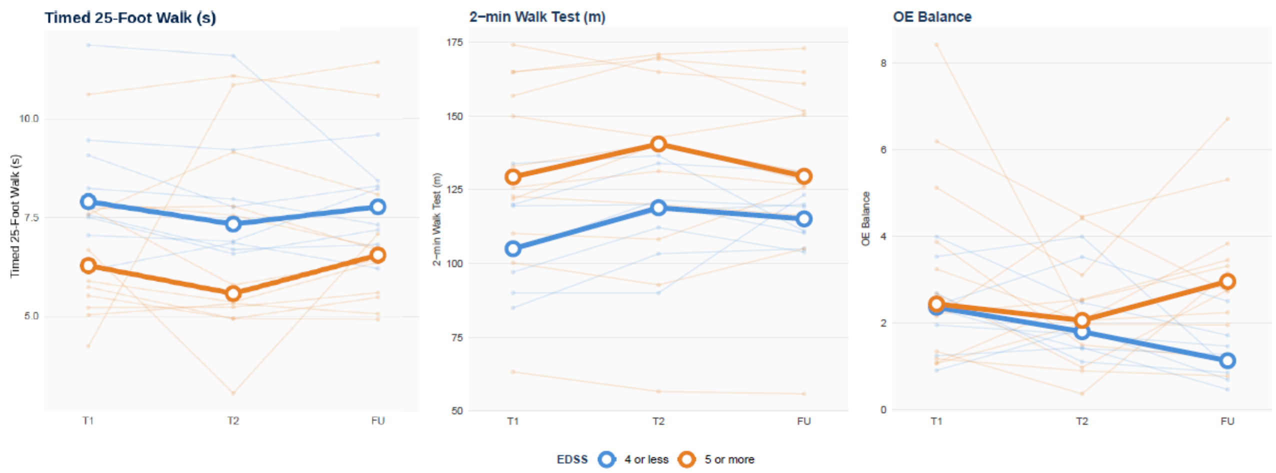
Timed 25-Foot Walk (s)
2–min Walk Test (m)
OE Balance
Timed 25-Foot Walk (s)
2–min Walk Test (m)
OE Balance
T1
T2
FU
EDSS
4 or less
5 or more

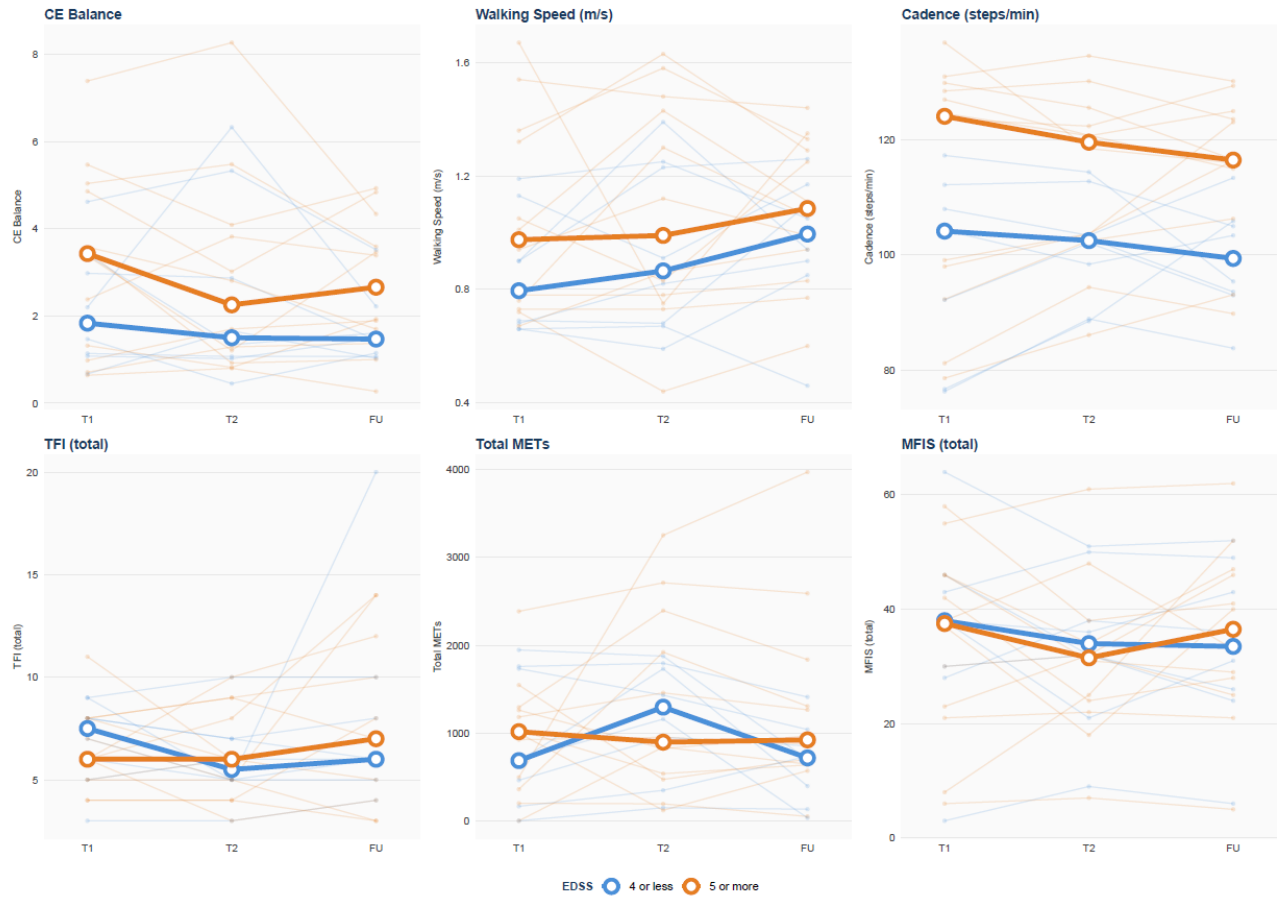
CE Balance
Walking Speed (m/s)
Cadence (steps/min)
TFI (total)
Total METs
MFIS (total)
CE Balance
Walking Speed (m/s)
Cadence (steps/min)
TFI (total)
Total METs
MFIS (total)
T1
T2
FU
EDSS
4 or less
5 or more

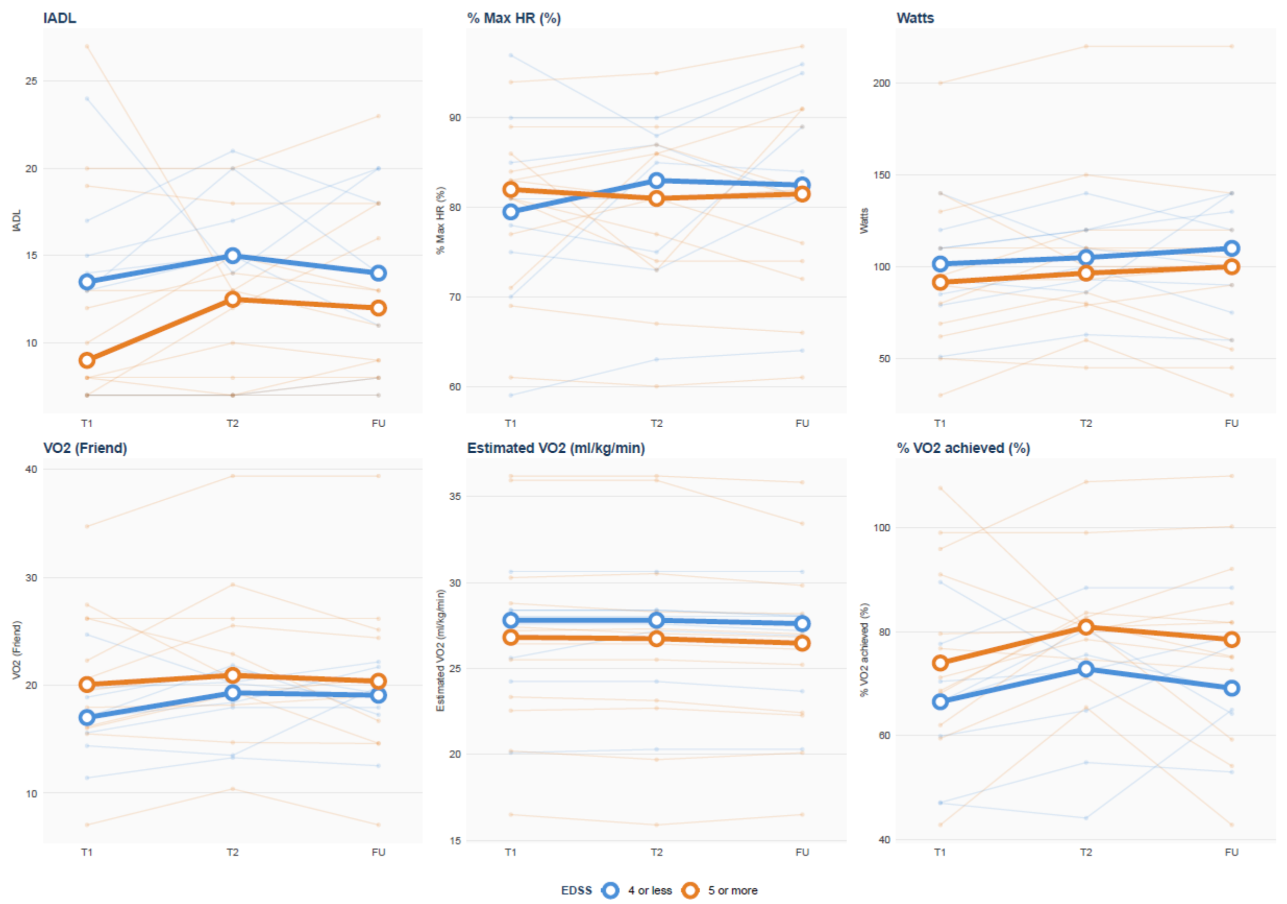
IADL
% Max HR (%)
Watts
VO2 (Friend)
Estimated VO2 (ml/kg/min)
% VO2 achieved (%)
T1
T2
FU
EDSS
4 or less
5 or more

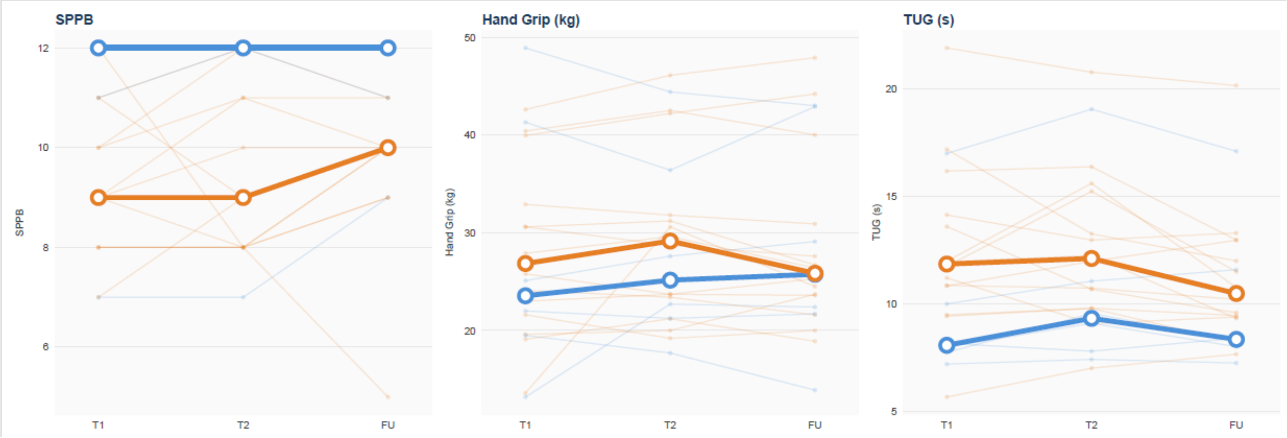
SPPB
Hand Grip (kg)
TUG (s)
T1
T2
FU

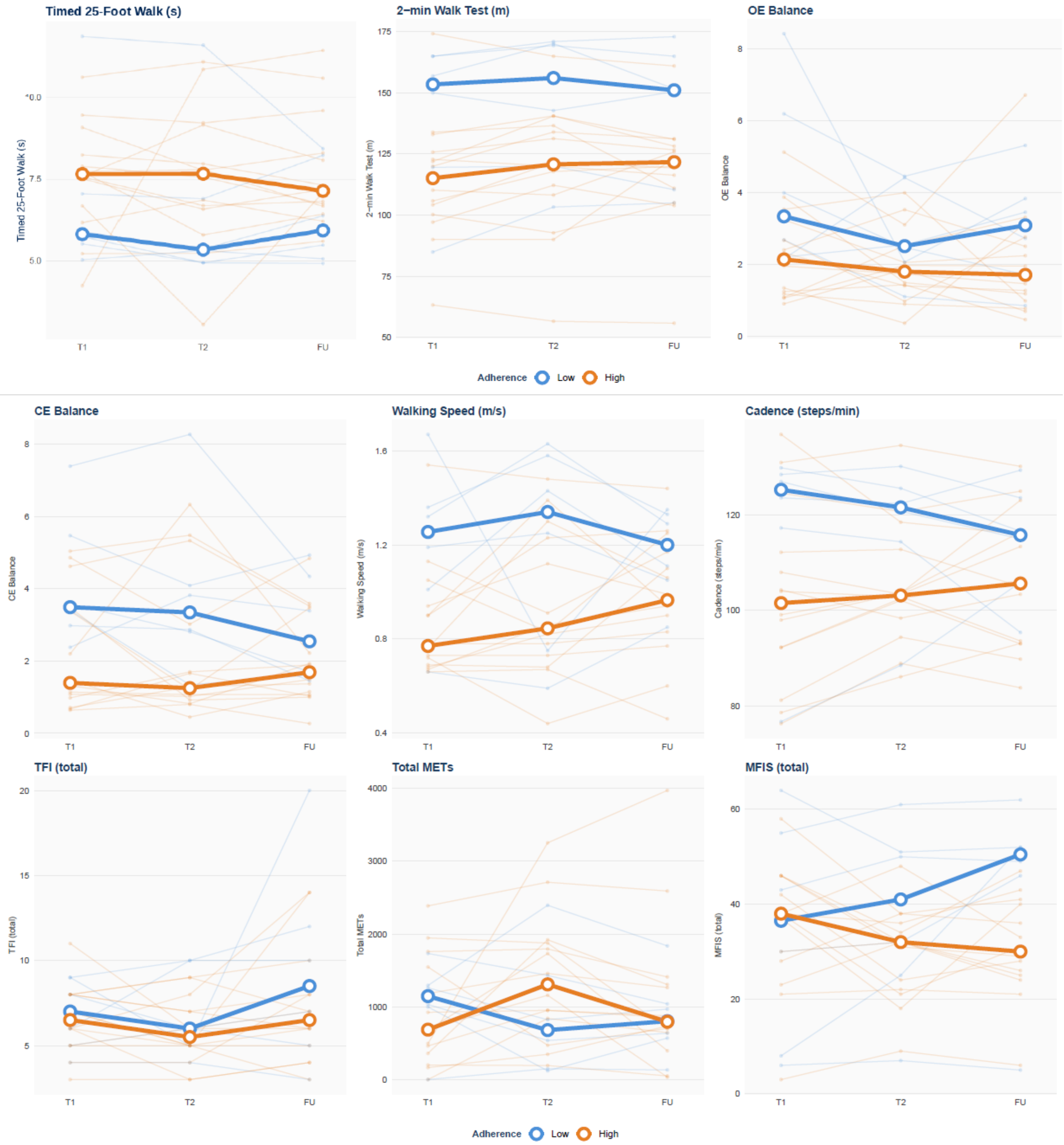

Timed 25-Foot Walk (s)
Timed 25-Foot Walk (s)
2−min Walk Test (m)
2−min Walk Test (m)
OE Balance
OE Balance
T1
T2
FU
Adherence
Low
High
CE Balance
CE Balance
Walking Speed (m/s)
Walking Speed (m/s)
Cadence (steps/min)
Cadence (steps/min)
TFI (total)
TFI (total)
Total METs
Total METs
MFIS (total)
MFIS (total)
Adherence
Low
High

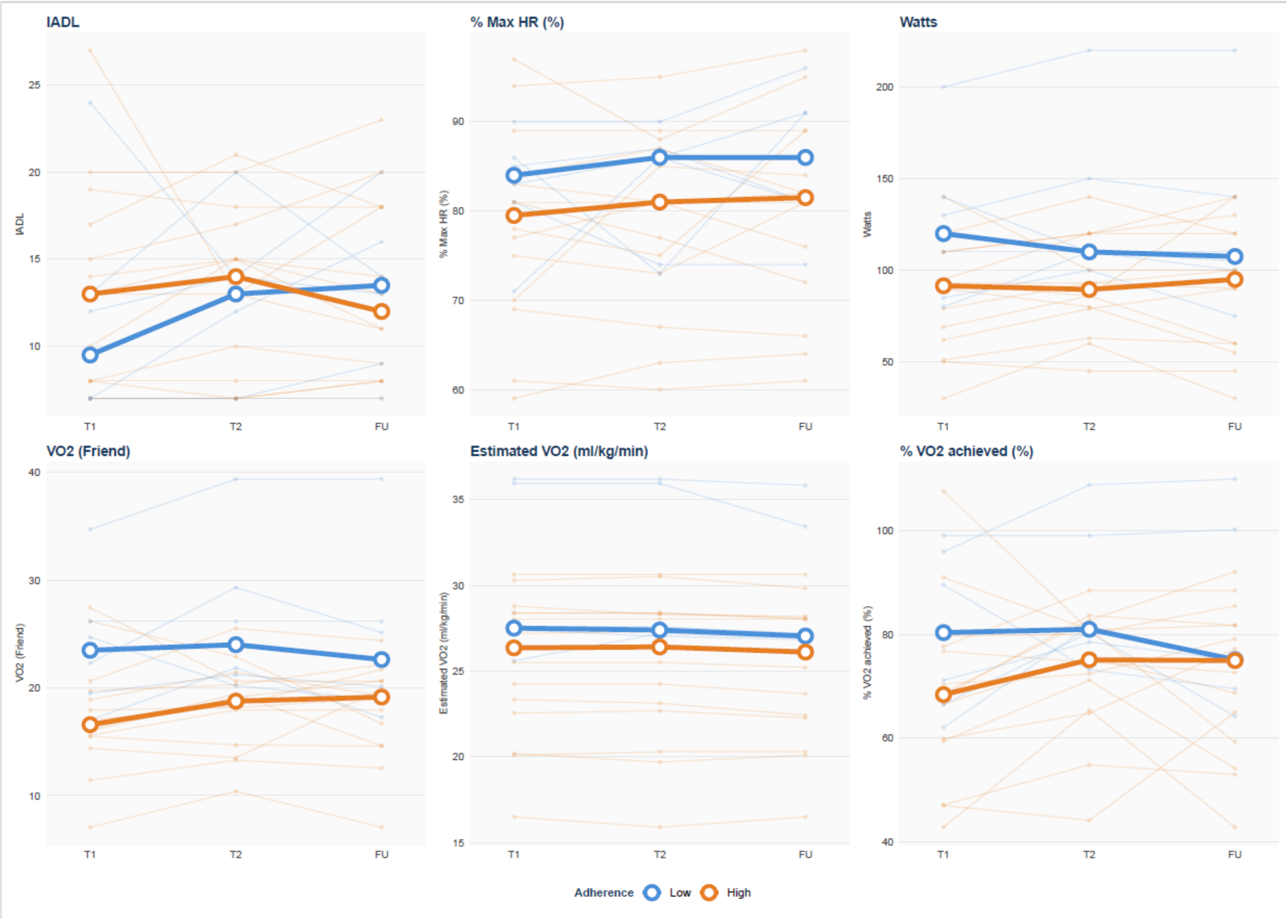
IADL
% Max HR (%)
Watts
VO2 (Friend)
Estimated VO2 (ml/kg/min)
% VO2 achieved (%)
T1
T2
FU
Adherence
Low
High
